# Superior effects of high-intensity interval eccentric cycling training on neuromuscular adaptations with similar aerobic adaptations to concentric cycling

**DOI:** 10.1007/s00421-025-05848-5

**Published:** 2025-08-08

**Authors:** Marcin Lipski, Joanne Trezise, Chris R. Abbiss, Kazunori Nosaka

**Affiliations:** 1 Alatus Wellbeing, Health and Performance, Invercargill, Southland 9874 New Zealand; 2 Human Performance Centre, Southern Institute of Technology, Te Pūkenga, Invercargill, Southland New Zealand; 3 School of Medical and Health Sciences, Edith Cowan University, Joondalup, Australia

**Keywords:** Lengthening contraction, VO_2peak_, Peak power output, HIIT, Eccentric training, Muscle cross sectional area

## Abstract

**Purpose:**

We compared the effects of high-intensity interval eccentric (EC) versus concentric cycling (CC) training on aerobic capacity, muscle function and morphology.

**Methods:**

Healthy men (19–56 y) performed EC (n = 9) or CC (n = 8) training twice a week for 8 weeks. The training progressed from 5 × 2-min intervals with 1-min rest to 7 × 2-min intervals with 30-s rest. EC and CC were matched for perceived effort, and progressed from 30 to 36% of concentric peak power output (PPO_10s_) for CC and from 45 to 70% PPO_10s_ for EC. Changes in peak oxygen consumption (VO_2peak_), incremental concentric PPO (PPO_inc_), 6-min walking distance (6 MW), 10 s concentric PPO (PPO_10s_), maximal voluntary isometric contraction knee extensor strength (MVC), countermovement (CMJ) and squat jump height (SJ), quadriceps cross-sectional area (CSA), and fascicle length (FL) and pennation angle (PA) of vastus lateralis were compared between EC and CC.

**Results:**

Greater (P < 0.05) changes in PPO_10s_ (EC: 26.9 ± 10.5% vs. CC: 8.9 ± 8.0%, Hedges’*g* = 2.03), CMJ (3.9 ± 1.8 vs. − 3.3 ± 7.4%, *g* = 1.46), SJ (7.4 ± 4.7% vs. − 2.3 ± 4.4%, *g* = 2.26), and CSA (6.1 ± 4.7 vs. 0.1 ± 3.8%, *g* = 1.48) were observed after EC than CC. No significant differences between EC and CC were found for changes in VO_2peak_ (3.7 ± 3.9 vs. 6.6 ± 6.9%, g = -0.55), PPO_inc_ (6.0 ± 4.2 vs. 6.4 ± 4.6%, g = − 0.11), 6 MW (6.0 ± 4.2 vs. 6.4 ± 4.6%, g = -1.03) and MVC (12.5 ± 13.3 vs. 6.2 ± 8.3%, g = 0.59). FL and PA did not show significant changes after EC and CC.

**Conclusion:**

EC was more effective than CC for improving several markers of muscle function. High-intensity interval eccentric cycling appears to be suitable for simultaneously improving strength and endurance.

**Graphical abstract:**

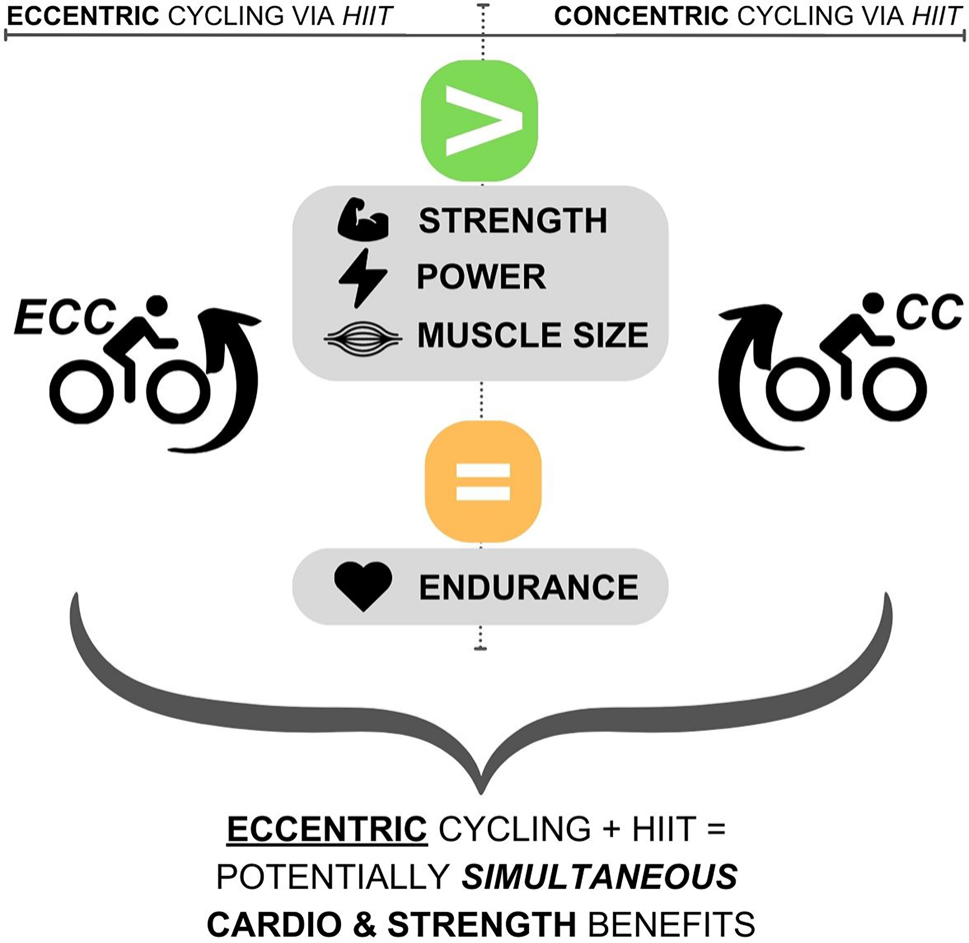

## Introduction

Eccentric cycling, in which knee and hip extensor muscles perform eccentric (lengthening muscle) contractions when resisting to backward rotating pedals, is seen more due to an increase in commercially available machines. Eccentric cycling (EC) is 40–60% more oxygen cost efficient than conventional or concentric cycling (CC) at the same power output (Abbott et al. 1952; Peñailillo et al. 2013). Much greater power outputs are produced during EC than CC, with recreationally trained individuals participating in multiple sports reaching 1700 W during sprints of upright EC (Brughelli and Van Leemputte 2013) and up to almost 3000 W during sprints of recumbent EC (Green et al. 2018). We have reported that when performed until exhaustion in an incremental test, peak power was up to 53% greater in EC than CC (Lipski et al. 2018). However, the potential training benefits of this high-power output during EC have not been extensively examined.

Leong et al. (2013) reported a large increase in training power of up to 280% (157–442 W) after 5–10 min of EC training performed two sessions per week for eight weeks in young healthy individuals. Thus, it appears that EC may provide a potent training stimulus resulting in rapid and large improvements in muscle function. However, the majority of previous studies investigating the effects of EC training have adopted relatively constant pace exercise and low training intensities such as 60% maximal heart rate or 20–60% concentric peak power output (Lastayo et al. 1999, 2000; Mueller et al. 2009, 2011). Despite this, it has been found that 6–11 weeks of EC training improves muscle function and morphology (Lastayo et al. 1999, 2000, 2003; Lindstedt et al. 2002; Leong et al. 2013) and aerobic capacity particularly in clinical populations (Rooyackers et al. 2003; Meyer et al. 2003; Marcus et al. 2009; Gremeaux et al. 2010; Lastayo et al. 2011; Besson et al. 2013; Lattouf et al. 2021). However, EC training effects on cardiovascular parameters in healthy, non-athletic populations have been largely unexplored.

Low-intensity and high-volume eccentric contractions, such as those performed in downhill walking (Zeppetzauer et al. 2013) and descending stair walking (Chen et al. 2017), have been shown to improve respiratory function. Additionally, EC can elicit a load on central cardiovascular function, resulting in a greater cardiac output (Dufour et al. 2004) and blood pressure (Rakobowchuk et al. 2018) when compared with CC. Thus, it is possible that EC training can also induce adaptations in the cardiovascular system, when it is performed in a format of a high-intensity interval training.

Performing concentric cycling high-intensity interval training (HIIT) has been shown to be effective in improving maximal oxygen consumption and exercise performance (Gibala et al. 2012), insulin sensitivity (Babraj et al. 2009), and an array of markers associated with mitochondrial biogenesis and function (Burgomaster et al. 2005). However, it appears that the effects of HIIT on muscle strength and muscle size are limited (Ross and Leveritt 2001). HIIT is an efficient mode of exercise that requires a short exercise time, which is important since the time taken for exercise is a factor affecting the compliance to exercise (Kimm et al. 2006). Thus, a combination of EC and HIIT could induce both neuromuscular and cardiovascular adaptations.

Two studies have investigated the effects of high-intensity EC interval training with either 2-min (Paulsen et al. 2019) or 1 min intervals (Mavropalias et al. 2022). While both showed an increase in muscle size and strength, peak and average concentric power output during cycling sprints only improved in the latter study (Mavropalias et al. 2022). Interestingly, Paulsen et al. (2019) showed a decrease in endurance performance (i.e., maximal aerobic power output and 20 min time trial performance), in spite of high-intensity CC interval exercises being added directly after EC in each session. It may be that such a combination of stimuli is inadequate for improving endurance performance, or even detrimental. Thus, further studies are required to investigate the effects of isolated high-intensity interval EC training on both muscle and endurance function.

It may be that HIIT EC provides concurrent stimulus to neuromuscular and cardiopulmonary systems. However, to the best of our knowledge, no previous study has investigated whether HIIT EC produces greater adaptations in these systems when compared with concentric HIIT cycling. It is plausible to assume that the greater power output that can be achieved during HIIT EC increases muscle strength and improves incremental peak power output.

Therefore, the present study compared the effects of EC and CC in a HIIT design on aerobic capacity, muscle morphology and muscle function. It was hypothesized that changes in the muscle cross-sectional area and strength would be greater after 8 weeks of HIIT EC, while changes in VO_2peak_ would be greater after HIIT CC. However, due to the greater functional improvements after HIIT EC, changes in concentric sprint and incremental peak power output would be similar between the two.

## Methods

### Participants

A generally active population with a wide age bracket was recruited to examine the effects of an EC versus CC HIIT design on muscle and aerobic parameters. The sample size is justified based upon an estimated effect size of 0.3 for a possible difference between EC and CC for changes in maximal isometric strength based on a previous study (MacMillan et al. 2017). This showed that a total of 14 participants were necessary with the power of 0.8 and α level of 0.05. Considering a possible estimation error, a total of 18 men who were physically active (performing exercise or sports at least once a week) were recruited to participate in the study. None of the participants had performed any specific or structured eccentric exercise, beyond those performed in daily activities (e.g., descending stairs), in the 6 months prior to the study. Participants reported that they were not under any medication nor had any history of lower limb musculoskeletal injuries. They were instructed to refrain from exercise, alcohol and caffeine intake in the 48 h prior to each testing session. Participants were fully informed of the requirements and risks associated with the study and provided a written informed consent before participation in the study in accordance with the institutional Human Research Ethics Committee that approved this study, and Declaration of Helsinki.

The participants were matched by VO_2peak_ and then one from each pair was place in either the EC or the CC training group. One participant in the CC group had to withdraw during the study due to a personal reason, thus the number of participants included in the results was nine for EC, and eight for CC. Their mean ± SD (range) age, body mass and height were 34.9 ± 12.5 (19–56; EC 26–56; CC 19–56) y, 88.7 ± 16.2 (69.6–135.2; EC 75.8–113.5; CC 69.6–135.2) kg, and 178.4 ± 6.9 (169–191; EC 169–192; CC 170–191) cm, respectively. Age, body mass and height were not significantly different between the groups.

### Study design

All participants performed either interval eccentric (EC) or concentric cycling (CC) training twice a week for 8 weeks. Baseline measurements were taken 3 to 7 days before the first training session, and all measurements were repeated 2–5 days following the last training session. While 5 days between the end of intervention and post-training assessments were long, previous studies in which high-intensity eccentric cycling was performed showed some delayed improvement in performance (Leong et al. 2013). It was also assumed that the effects of training were sustained for 5 days, thus the range of interval (2–5 days) did not affect the results.

The outcome measures included 6 min walking distance (6 MW), countermovement jump height (CMJ), squat jump height (SJ), maximal voluntary isometric contraction strength of the knee extensors (MVC), peak oxygen consumption (VO_2peak_), incremental peak power output (PPO_inc_), quadriceps cross-sectional area (CSA), fascicle length (FL), pennation angle (PA), and concentric sprint peak power output (PPO_10s_) in this order.

The first day of the testing included 6 MW, CMJ, SJ, MVC and an incremental cycling test to exhaustion. There were 15 min of passive rest between the 6 MW and the jump tests, as well as between the jumps and the MVC, and also between the MVC and the incremental cycling test. As participants were all active and regular participants in sports, it was assumed that the multiple physical assessments would be unlikely to be affected by fatigue or inflammation. The second day of testing covered CSA, FL and PA measurements of the quadriceps. Participants then completed two familiarization sessions to practice EC (EC group) or experience concentric cycling (CC group), on an isokinetic cycling ergometer (Grucox Isokinetic Ergometer, South Africa). A 10-s maximal CC sprint was performed at the start of the first familiarization session to measure PPO_10s_.

The first familiarization session then included 8-min EC at 30% of PPO_10s_ for the EC group, and 3 repetitions of 1-min CC at 30% of PPO_10s_ with 30-s rest between repetitions for the CC group. The second familiarization session consisted of 10-min EC at 35% of PPO_10s_ for EC and 2 repetitions of 2 min CC at 30% of PPO_10s_ with 1-min rest between repetitions for CC. Although participants were active in the rest periods, they were asked in both groups to apply minimum force to the moving pedals, as the isokinetic ergometer was continuously moving the pedals at 60 rpm. The familiarization sessions were completed 1 to 3 days before the first training session. All participants had 16 training sessions detailed below (twice a week for 8 weeks). Changes in the outcome measures from baseline to post-training were compared between EC and CC groups.

### Training protocols

**Fig. 1:**
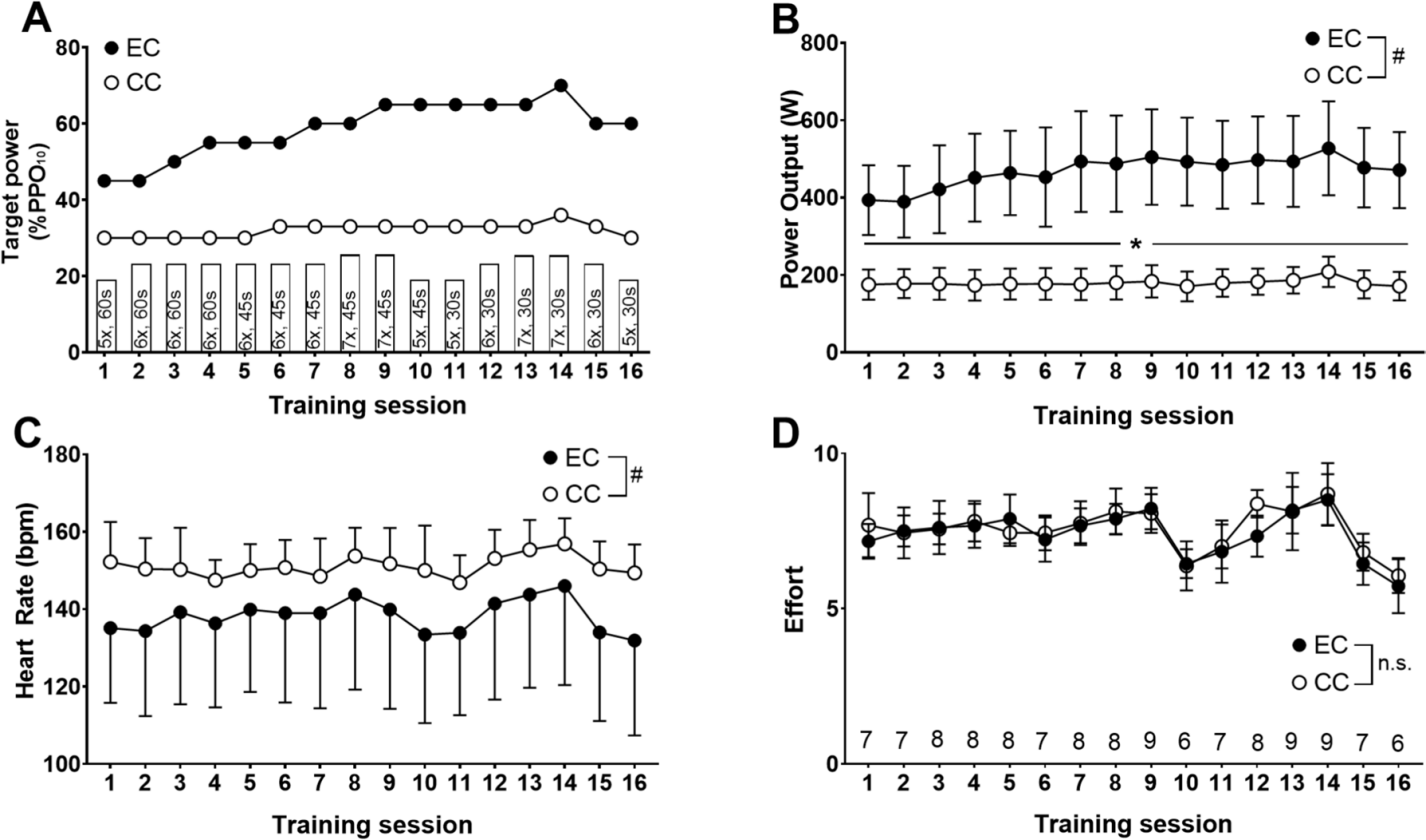
Training program based on the percent of concentric 10-s sprint peak power output (PPO) (**A**), and actual average power output (**B**), heart rate (**C**), and rating of perceived effort (**D**) over 16 training sessions for eccentric (EC) and concentric (CC) cycling training groups. A: the target power output is shown as the percentage of the peak power output achieved during a concentric 10-s sprint. The duration of cycling was always 2 min per repetition, while the rest between repetitions was initially 60 s, and progressed to 45 s then 30 s. For each training session, the number of repetitions and rest time for each session were indicated in the bar (e.g. 5 repetitions, 60 s interval: 5×, 60 s). D: Target rating of perceived effort for each session is shown above the x-axis. ^#^significant (P < 0.05) group effect, *significant (P < 0.05) difference between groups

All participants performed all sessions on the same cycling ergometer (Grucox Isokinetic Ergometer, South Africa) at a constant cadence of 60 revolutions per minute (RPM) in an isokinetic mode for forward (CC) or backward rotations (EC). All training sessions were performed in intervals, and the training intensity and volume were periodised. Cycling duration was maintained for 2 min. However, the rest periods, target power output and effort were manipulated for each session (Fig. 1A). Due to the different physiological characteristics between EC and CC (Abbott et al. 1952; Peñailillo et al. 2013), both groups were matched only for training time and perceived effort. This type of matching was chosen to enhance practical application of the findings, but also maximise intensity and possible cardiovascular stimulus during EC and CC.

Due to the high-intensity nature of the training protocol, the periodization and increases in training intensity were less pronounced during CC than EC (Peñailillo et al. 2017; Mavropalias et al. 2020, 2022). For both groups, the exercise intensity was adjusted for each session based on the individual’s rating of perceived effort (scale of 0 to 10, with 0.5 increments) which was recorded after each interval. Participants were asked, “*How much mental and physical effort do you require right now to achieve and maintain the target power output?*”. Participants were also asked to rate their perceived effort for the training 1 min after each training session. The training sessions were periodised to have double peaks of volume in training sessions 8 and 9, and 13 and 14, with a decrease in volume in training sessions 10 and 11, and 15 and 16 (Fig. 1A). The intensity of training was manipulated to ensure that perceived effort was between 7 and 9 in all sessions, except for the final session where perceived effort was 6 (Fig. 1D). This resulted in the target power output being increased from 45 to 70% of PPO_10s_ for EC, and from 30 to 36% of PPO_10s_ for CC. When the reported rating of perceived effort was above or below the target effort, the target power output was adjusted for the following repetitions. Both groups performed 5 repetitions of 2-min cycling with 1-min rest in the first session, and it increased to 7 repetitions of 2 min with 30 s rest (Fig. 1A). Although participants were active in the rest periods, they were asked in both groups to apply minimum force to the moving pedals, as the isokinetic ergometer was continuously moving the pedals at 60 rpm.

### Outcome measures

#### Aerobic capacity

The incremental test was performed to establish concentric PPO_inc_ and VO_2peak_ on a Velotron cycling ergometer (RacerMate, Inc., Seattle, Washington USA). Prior to the incremental test, participants completed a 3 min concentric warm-up at 50 W and 60 rpm. The incremental test protocol began at 75 W and increased by 25 W every minute until volitional exhaustion, and verbal encouragement was provided during the final stages of the test. Participants were able to self-select their cadence during the CC test, and the test was terminated when the cadence dropped below 60 rpm for more than 30 s. The PPO_inc_ was calculated based on the last stage completed and pro rata of any uncompleted stages (i.e., 25 W/60 s × time completed in the last stage). Expired gases were measured breath-by-breath and averaged every 15 s using a metabolic cart (TrueOne 2400 metabolic cart, ParvoMedics, Sandy, USA). The metabolic cart was calibrated before each test using gases of known concentrations and a 3-L syringe (5530 series, Hans Rudolph Inc., Shawnee, Kansas, U.S.A.). The VO_2peak_ was the highest value in any 15-s interval. Heart rate was recorded using (S610, Polar, Finland), and maximal heart rate was noted as the highest heart rate observed in the test.

A 6-min walk test was performed indoors between two cones placed 20 m apart with a mark every 5 m. Participants were instructed to start at the first cone, walk to the second cone 20 m away, turn around and repeat it for 6 min. For the turn participants were required to place any foot on the line drawn on the floor beside the cone. Any technique for the change of direction was allowed as long as one foot touched the line. Participants were strictly instructed to “walk as far as possible”, not run, and they were provided with verbal encouragement. The remaining time was indicated at 4, 3, and 1 min. The total walking distance achieved was recorded.

#### Muscle morphology

CSA, FL and PA were assessed via extended-field-of-view B-mode ultrasonography (10 MHz linear-array: Aloka SSD-alpha10, Aloka Co., Ltd., Tokyo Japan) with a 60-mm linear transducer. For all 3 measurements, the images were obtained with the participant lying in a supine position rested for at least 10 min (Noorkoiv et al. 2010). At the distance of 33% (distal), 50% (mid) and 66% (proximal) from the most lateral point of the patella to the top of the greater trochanter of the femur, 3 axial perpendicular lines were marked. Images were taken while applying consistent pressure to the probe avoiding muscle compression. Ultrasound transmission gel was applied. For the CSA scans, the probe was moved on the marked lines transversely over the thigh while taking a single continuous view. Three images were taken for each site and were analysed with digitising software (ImageJ 1.41, Wayne Rusband, National Institutes of Health, USA) for cross-sectional area of all compartments of the quadriceps femoris at the distal, mid and proximal sites. All cross-sectional area measures were analysed in triplicate by the same investigator. Total CSA for each site was calculated by taking the sum of CSA for all quadriceps compartments at the relevant site (Noorkoiv et al. 2010).

Scans for FL and PA were taken from the vastus lateralis (VL) at a distance of 50% between the lateral centre of the knee joint and the top of the greater trochanter of the femur. Each point was marked with a 4-mm wide double-sided adhesive tape to provide a shadow in the ultrasound image. FL and PA were analysed using digitising software (ImageJ 1.41, Wayne Rusband, National Institutes of Health, USA). The fascicle at 50% of the distance from the deep aponeurosis to the superficial aponeurosis of vastus lateralis, marked by the shadow produced by the adhesive tape, was analyzed for length. For the analysis of the pennation angle for the vastus lateralis the line drawn for the fascicle length at the deep aponeurosis to the shadow at 50% of the fascicle length was used in conjunction with a second line. To avoid the slightly greater fascicle curvature at its insertion, the second line was drawn parallel to the deep aponeurosis and 3 mm above the starting point of first line used for the fascicle length. The FL and PA measures were analysed in triplicate, and the average of the 3 measures was used for further analysis.

#### Muscle function

Following a 2 min self-selected warm-up and 30 s at 60 W, participants performed a 10 s seated cycling sprint at 60 rpm on the isokinetic cycling ergometer (Grucox Isokinetic Ergometer, South Africa) from a stationary start. Verbal encouragement was provided during all sprints. The sampling frequency of the ergometer was 10 Hz, and PPO_10s_ was the highest power output reached in the 10 s.

Maximal voluntary isometric contraction (MVC) torque of the knee extensors was measured using a custom-made chair with a load cell (Xtran S1W, Applied Measurements, Melbourne, Australia) (Peñailillo et al. 2014). The measurement was taken on the right leg at 70° knee flexion. Prior to the measurements participants performed a 5 min warm-up on a cycling ergometer (Monark 828E, Monark Exercise AB, Vansbro, Sweden) at 15% of their PPO_inc_ and 60 rpm. The warm-up was followed by three 3 s submaximal isometric knee extensions 50, 50 and 80% of a maximal effort, each seperated by 1 min passive rest. Three maximal voluntary 3 s isometric contractions of the knee extensors were performed, with a 1-min passive rest between trials. Participants were advised to contract “as fast and as hard as possible”, and trials with any visual countermovement were disregarded and repeated. The torque data was sampled at a frequency of 1000 Hz, and a digital zero-phase lag finite impulse response low-pass filter with a cut-off frequency of 14 Hz was applied. The attempt with the greatest peak torque value was used for further analysis. Verbal encouragement was provided during all measurements.

Countermovement and squat jumps were performed on a force plate (9290AD; Kistler Instruments, Winterthur, Switzerland). Participants performed all jumps at a self-chosen squat depth but with less than 90° knee flexion while hands were placed on their hips. For the countermovement jump, participants were asked to jump as high as possible from an upright standing position on a count of 3. During the squat jump, participants were asked to squat down, remain in the squat position for 3 s and then jump without any countermovement. Vocal encouragement was provided during both jumps. First, participants performed 3 countermovement jumps followed by 3 squat jumps. Participants freely choose their rest, but no more than 2 min was allowed between jumps. Maximal jump heights were measured based on the flight time using computer software (MARS 2.1, Kistler Instruments, Winterthur, Switzerland), and the highest value was used for further analysis. The coefficient of variation (CV) across the trials for MVC, CMJ and SJ was 3.2, 1.9 and 1.8%, respectively.

### Statistical analyses

**Fig. 2:**
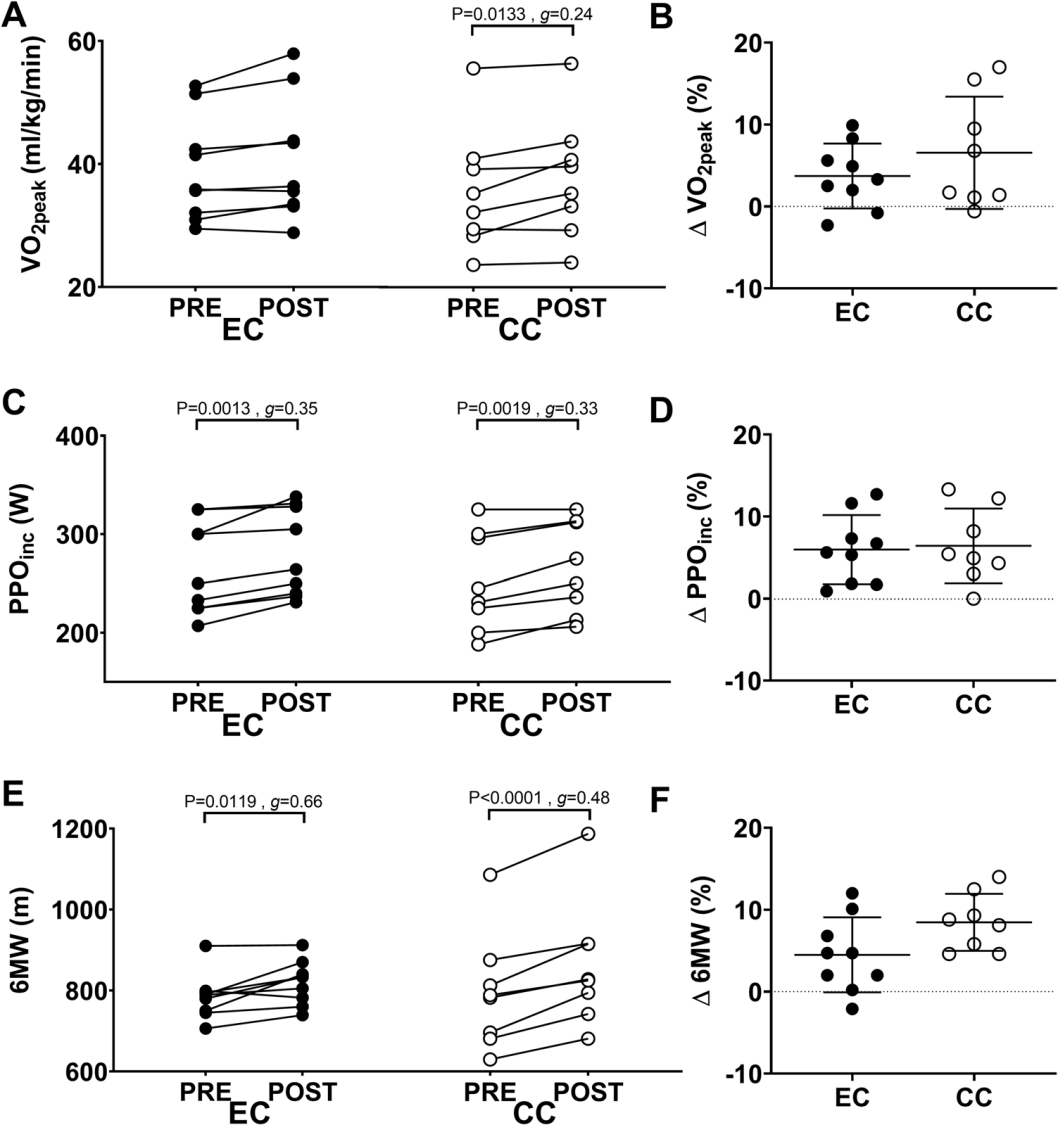
Comparison between eccentric (EC) and concentric (CC) cycling training groups for changes in peak oxygen consumption (VO_2_peak) (panel **A**, **B**), peak power output achieved in an incremental concentric cycling test (PPO_inc_) (panel **C**, **D**) and distance covered in a 6-min walking test (6 MW) (panel **E**, **F**). For each figure, individual data are plotted for changes in the variable from before (pre) to after training (post) on the left side (**A**, **C**, **E**), and percentage changes from pre- to post-training, and the average (long line) ± SD (short lines) on the right side (**B**, **D**, **F**). Effect size (g) for the difference between EC and CC groups is also shown

**Fig. 3:**
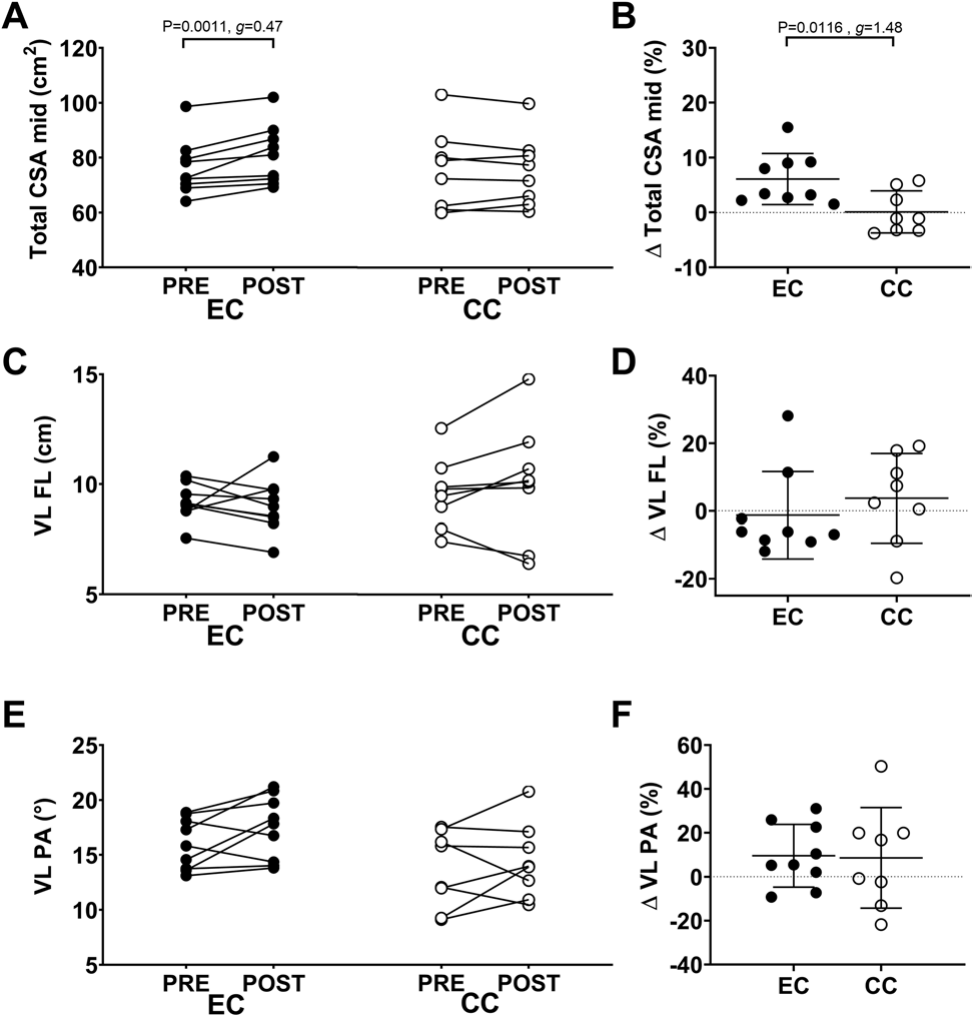
Comparison between eccentric (EC) and concentric (CC) cycling training groups for changes in quadriceps cross sectional area (CSA) (panel **A**, **B**), fascicle length of the vastus lateralis (FL) (panel **C**, **D**) and pennation angle of the vastus lateralis (PA) (panel **E**, **F**). For each figure, individual data are plotted for changes in the variable from before (pre) to after training (post) on the left side (**A**, **C**, **E**), and percentage changes from pre- to post-training, and the average (long line) and ± 1SD (short lines) on the right side (**B**, **D**, **F**). Effect size (g) for the difference between EC and CC groups is also shown

**Fig. 4:**
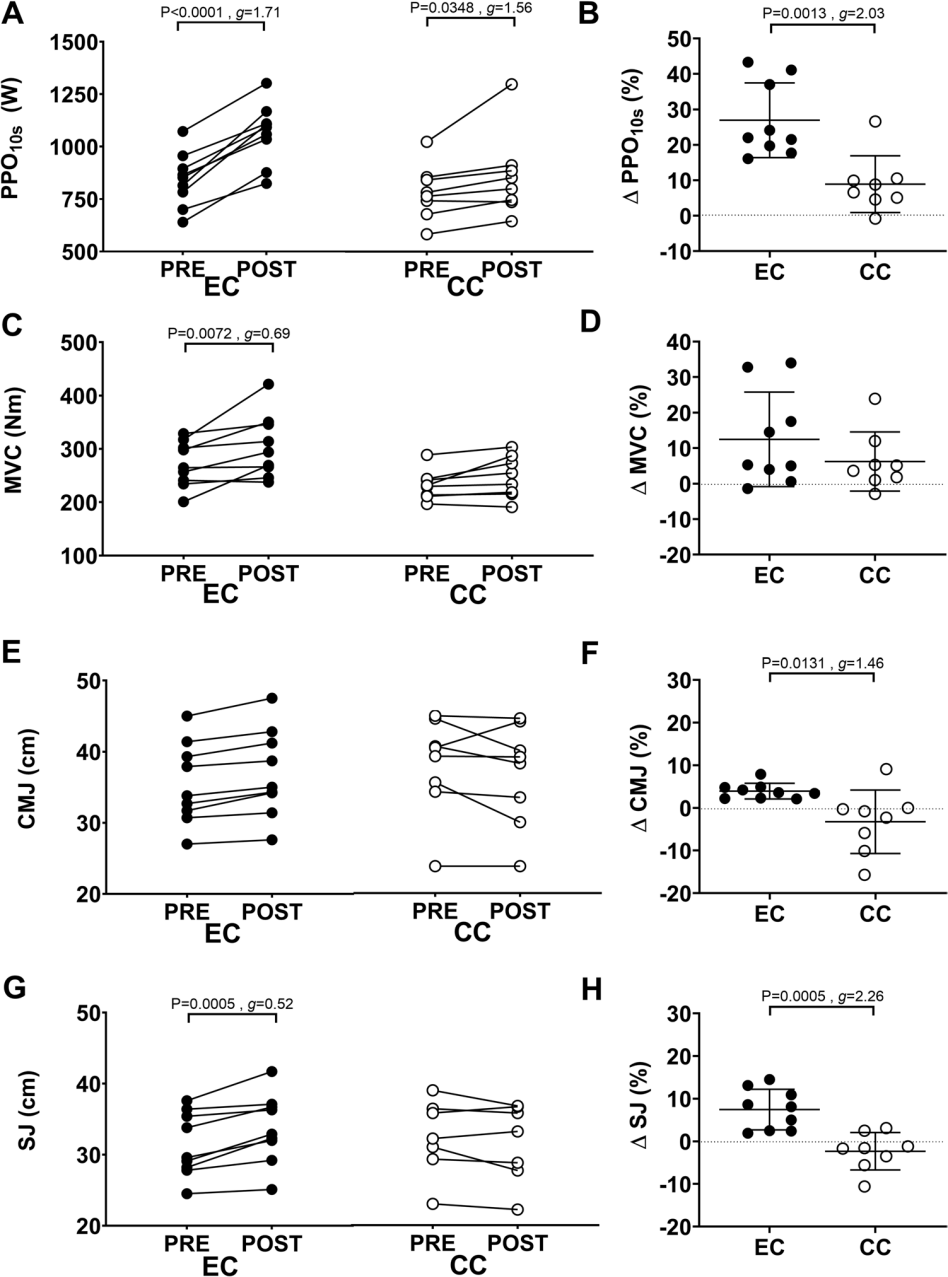
Comparison between eccentric (EC) and concentric (CC) cycling training groups for peak power output achieved during a 10-s sprint (PPO10s) (panel **A**, **B**), maximal voluntary isometric strength of the knee extensors (MVC) (panel **C**, **D**), countermovement jump height (panel **E**, **F**) and squat jump height (**G**, **H**). For each figure, individual data are plotted for changes in the variable from before (pre) to after training (post) on the left side (**A**, **C**, **E**, **G**), and percentage changes from pre- to post-training, and the average (long line) ± SD (short lines) on the right side (**B**, **D**, **F**, **H**). Effect size (g) for the difference between EC and CC groups is also shown

A two-way repeated measures analysis of variance (ANOVA) was used to compare the changes in the outcome measures from before to after the 8 weeks of training between EC and CC groups. When a significant interaction or time effect was found, Sidak´s post hoc analysis was performed. As the individual changes were of interest, independent t-tests were included with Figs. 1, 2, 3 and 4 to which compare the percentage change in variables between EC and CC. Hedges’ *g* was used to calculate the effect size for the magnitude of the changes in of variables within and between groups, and was interpreted as small effect (*g* = 0.2), medium effect (*g* = 0.5) and large effect (*g* = 0.8). Pearson’s correlation coefficients were also calculated to assess relationships between changes in the training intensity or changes in concentric sprint peak power output in 10 s (PPO10s) and changes in outcome variables for the EC and CC group separately. The significance was set at P < 0.05 for all analyses and all statistical analyses were performed using GraphPad statistical package (Prism version 7.02, GraphPad Software, La Jolla, California, USA).

## Results

### Training

Power output, heart rate and rating of perceived effort for each training session are presented in Fig. 1B–D. Power output during all training sessions was greater (P < 0.0001) during EC than CC. There was no difference between groups for any session in heart rate (P ≥ 0.53) and sessional rating of perceived effort (P ≥ 0.06). However, average heart rate across all training sessions was greater (P < 0.0001) for CC (151 ± 8 bpm) than EC (138 ± 22 bpm).

### Aerobic capacity

Significant time effects were found for changes in VO_2peak_, PPO_inc_, and 6 MW. The increase in VO_2peak_ from pre- to post-training did not reach significance for EC (P = 0.0532, *g* = 0.19), but was significant for CC (P = 0.0133, *g* = 0.24; Fig. 2A). The magnitude of the VO_2peak_ change was not significantly different (P = 0.3052, *g* = -0.55) between EC (3.7 ± 3.9%) and CC (6.6 ± 6.9%) (Fig. 2B). PPO_inc_ increased significantly following training in both EC (P = 0.0013; *g* = 0.35) and CC (P = 0.0019, *g* = 0.33) (Fig. 2C). The magnitude of increase in PPO_inc_ was not different (P = 0.8323) between EC (6.0 ± 4.2%) and CC (6.4 ± 4.6%) (Fig. 2D). 6 MW distance increased following training in both EC (P = 0.0119, *g* = 0.66) and CC (P < 0.0001, *g* = 0.48) (Fig. 2E), and the magnitude of the increase was not different (P = 0.0647) between EC (4.5 ± 4.6%) and CC (8.5 ± 3.5%) (Fig. 2F).

### Muscle morphology

Significant interaction effects were found for changes in CSA measured at distal, mid and proximal sites. CSA at the distal, mid and proximal site increased from pre- to post-training following EC (P < 0.0001,* g* = 0.82; P = 0.0011,* g* = 0.47; P < 0.0001,* g* = 0.6; respectively), but not CC (P = 0.787, *g* = − 0.06; P = 0.9623, *g* = 0.02; P = 0.8268, *g* = − 0.04; respectively) (Fig. 3A-B for mid site CSA). The percentage change in total CSA at the distal (EC: 10.6 ± 7.7% vs. CC: 1.3 ± 2.6%), mid (EC: 6.1 ± 4.7% vs CC: 0.1 ± 3.8%) and proximal site (EC: 9.8 ± 5.5% vs CC: 1 ± 2%) was greater after EC than CC (P = 0.0054, *g* = 1.68; P = 0.0116, *g* = 1.48; P = 0.0007, g = 2.2; respectively).

Fascicle length of the vastus lateralis was not different from pre- to post-training in either group (P = 0.2989; Fig. 3C), and no difference in the changes was evident between groups (P = 0.4433, *g* = 0.4) (Fig. 3D). PA of VL was not different between pre- and post-training in either group (P = 0.5790) (Fig. 3E), and no significant difference between EC and CC groups was found (P = 0.9169, *g* = 0.06) (Fig. 3F).

### Muscle function

Interaction effects for PPO_10s_, MVC, CMJ, and SJ were significant. PPO_10s_ increased from pre- to post-training following EC (P < 0.0001, *g* = 1.71) and CC (P = 0.0348, *g* = 1.56; Fig. 4A). The magnitude of increase in PPO_10s_ (P = 0.0013; *g* = 2.03) was greater for EC (26.9 ± 10.5%) than CC (8.9 ± 8%; Fig. 4B). Compared with baseline, MVC increased for EC (P = 0.0072, *g* = 0.69) but not for CC (P = 0.3029, *g* = 0.47; Fig. 4C). However, no difference (P = 0.2712, *g* = 0.59) in the magnitude of change in MVC was found between EC (12.5 ± 13.3%) and CC (6.2 ± 8.3%) (Fig. 4D). No change in CMJ was observed following EC (P = 0.0955, *g* = 0.26) or CC (P = 0.1976; g = 0.19) (Fig. 4E). SJ increased following EC (P = 0.0005, *g* = 0.52) but not CC (P = 0.3055; *g* = 0.19) Fig. 4G). The magnitude of increase in CMJ (P = 0.0131, *g* = 1.46; EC: 3.9 ± 1.8%; CC: − 3.2 ± 7.4%) and SJ (P = 0.0005, *g* = 2.26; EC: 7.4 ± 4.7 CC: − 2.3 ± 4.4%) were both greater for EC than CC (Fig. 4F&H).

### Correlations between the training intensity or PPO10s and outcome variables

As shown in Table 1, a moderate relationship was evident only between the increase in training intensity and the increase in mid-thigh total quadriceps CSA (R^2^ = 0.46, P = 0.032) in the EC group.

**Table 1:** Pearson correlation analysis results between the changes in training power output or changes in concentric sprint peak power output in 10 s (PPO10s) and the changes in maximum voluntary isometric contraction strength of the knee extensors (MVC), cross-sectional area of quadriceps at mid-thigh (CSA), squat jump (SJ) and countermovement jump (CMJ)

	Variables							
	MVC		CSA		CMJ		SJ	
	R^2^	P	R^2^	P	R^2^	P	R^2^	P
Change in training intensity								
EC	0.32	0.087	0.46	0.032*	0.00	0.900	0.06	0.506
CC	0.02	0.734	0.04	0.650	0.196	0.320	0.26	0.246
Change in PPO10s								
EC	0.004	0.862	0.25	0.170	0.11	0.394	0.30	0.099
CC	0.04	0.685	0.29	0.209	0.30	0.201	0.41	0.123

*Indicates a significant (P < 0.05) correlation

## Discussion

The present study compared the effects of HIIT eccentric (EC) and concentric cycling (CC) training on aerobic capacity, muscle morphology and muscle function. The magnitude of changes in 10 s peak power output during concentric cycling (PPO_10s_), cross-sectional area of the quadriceps muscle (CSA), and squat jump (SJ) height were significantly greater after EC than CC training, and the magnitude of changes in aerobic parameters were similar between EC and CC, but both EC and CC did not affect fascicle length and pennation angle of the quadriceps muscles. These partially supported the hypothesis that changes in the muscle cross-sectional area and strength would be greater after EC than CC, while changes in peak oxygen consumption (VO_2peak_) would be greater after CC than EC, but changes in concentric peak power would be similar between EC and CC.

The training intensity was matched between groups using rating of perceived effort in the present study, because it was previously shown that the rating of perceived effort may better represent training load during EC than rating of perceived exertion (Peñailillo et al. 2017). The matching of the training intensity between groups was successful (Fig. 1D), and across all sessions, target effort was achieved in both EC (97 ± 5%) and CC (98 ± 8%). Matching perceived effort resulted in a 35% increase in average power output during EC (392 ± 85 W to 530 ± 114 W) and 19% increase in average power output during CC (175 ± 39 W to 208 ± 39 W). Although the training was programmed to begin at 45% of PPO_10s_ and progress to 70% (Fig. 1A), the highest training power output among participants in EC ranged from 52 to 77% of PPO_10s_.

Inter-individual differences in the ability to produce high power outputs during HIIT EC appear to be dependent on factors such as eccentric muscle work exposure in the past, and eccentric coordination skill. In the present study, these inter-individual differences have also manifested itself in the larger SD for the target power output. Plus, these differences might have impacted the differences in average HR during the training sessions. Our previous study (Lipski et al. 2018) showed that heart rate responses were significantly different between eccentric cycling and concentric cycling such that the heart rate response during eccentric cycling was slower. Also, increases in heart rate per oxygen consumption and per time unit were significantly larger during eccentric than concentric cycling. As such, due to these mechanistic differences in how heart rate responds, the significant difference between groups in heart rate during the intervals was likely due to the differences in power outputs achieved, as well as their ability to control eccentric actions.

There was no significant difference in the magnitude of change in PPO_inc_, VO_2peak_ and 6 MW after training between groups, while both groups showed significant increases in the variables (Fig. 2). Furthermore, the average heart rate across all training sessions was greater during CC than EC, but when compared within each session there was no significant difference (Fig. 1C). Interestingly, some participants in the EC group had an average heart rate over 150 bpm during training, and showed 2.6% increase in VO_2peak_.

It appears that the cardiovascular load indicated by the heart rate was high enough to induce aerobic adaptations (Garber et al. 2011) for both EC and CC. The VO_2peak_ after CC increased by 6.6 ± 6.9%, which was lower than that observed in a previous study (Milanović et al. 2015) examining the benefit of HIIT CC (13 ± 7%)(Milanović et al. 2015). In the present study, the cadence fixed at 60 rpm and the work to rest ratio ranged from 2 to 4, which might have attenuated the increase in VO_2peak_ after CC training. In the study (Milanović et al. 2015), HIIT CC was performed on average for 81 ± 79 s at 99 ± 22 rpm with a work to rest ratio ranged from 0.1 to 2.

The magnitude of the increase in VO_2peak_ was not significantly different between EC and CC groups, although it should be noted that several participants in the CC group experienced large increases (Fig. 2B). Additionally, it has been reported that resistance training increases parameters associated with aerobic capacity (Beattie et al. 2014), and individuals with lower levels of aerobic capacity at baseline could specifically benefit from increases in lower limb strength and power production (Ozaki et al. 2013). Previous studies in which continuous EC training was performed for several weeks (Knuttgen et al. 1982; Lastayo et al. 2000) showed little or no increases in VO_2peak_. Thus, it is likely that the high-intensity interval protocol implemented in the present study worked to increase VO_2peak_.

A greater increase in muscle CSA was observed following EC than CC (Fig. 3AB), but no significant changes in FL and PA were observed following either EC nor CC training (Fig. 3CF). Previous studies that used continuous EC in a recumbent position have found greater increases in lean leg mass after eccentric than CC training (Lastayo et al. 2007; Gross et al. 2010). Other studies using continuous EC in a recumbent position also showed increases in muscle fibre CSA of VL by 40 to 60% (Fridén et al. 1983; Lastayo et al. 2000, 2003). Leong et al. (2013) also reported that EC in a recumbent position performed for 10 min continuously at 20–55% of peak power output achieved during a 6-s sprint, induced 13% increase in VL muscle thickness and 24% increase in rectus femoris muscle thickness. Elmer et al. (2010) found the contribution to power production from the knee and hip extensors to be 58 and 29% respectively, in a recumbent EC at a low intensity of 20% of concentric power output. It should be noted that the participants in the present study performed EC in the upright seated position at a much higher intensity (see Fig. 1). Furthermore, Kato et al. (2011) reported that during concentric cycling, torque production was different between upright and recumbent positions, which was also affected by joint angles. Therefore, it is possible that involvement of other muscles including hip flexors and extensors in the upright EC may have reduced the changes in muscle architecture. Further research is required to compare muscle activities between recumbent and upright eccentric cycling protocols. 

Although no statistically significant changes in FL and PA were observed after EC as a group, it is interesting that many participants in the EC group showed decreases in FL and increases in PA, and some participants in the CC group showed increases in FL and decreases in PA (Fig. 3). It seems possible that effects of EC on FL and PA were different from those of resistance exercise training in which a heavier load and a smaller number of contractions are used. It should be noted that the extended field of view method was used to measure FL and PA in the present study, which could have led to a different finding to the previous studies.

Franchi et al. (2017) in their review article have stated that FL increases after eccentric training and PA increases after concentric training, but none of the included studies in the review utilized EC. Reeves et al. (2009) reported 20% increase in FL after eccentric resistance training, and 35% increase in PA after concentric training, in which consisted of 14 weeks (3 times per week) of isotonic knee extensions and leg press (2 × 10 repetitions) at 80% of eccentric 5 repetition maximum (RM) for the eccentric, and at 80% of concentric 5RM for concentric training. Franchi et al. (2014, 2015) also showed that FL increased 5% or 12% after 4 weeks or 10 weeks of isotonic leg press training performed twice a week (4 × 8–10 repetitions per session) at 80% of eccentric 1RM, and PA increased 7% or 30% after 4 weeks or 10 weeks of the leg press training at 80% of concentric 1RM. In contrast, Leong et al. (2013) reported that continuous EC performed in a recumbent position for 5–10 min at 20–55% of peak power output during a 6-s concentric sprint performed twice per week for 8 weeks increased PA of the VL by 24%. Indeed, Reeves et al. (2009) and Franchi et al. (2017) stated that greater external loads, especially during eccentric muscle actions were necessary to induce architectural changes. Additionally, the contraction velocity in the previous studies was approximately 30°/s, which is much slower than the velocity during HIIT EC in this study (180°/s at 60 rpm). This might contribute to the different adaptations in FL and PA between eccentric resistance exercise versus EC training. Additionally, Franchi et al. (2014, 2015) also found increases in FL after concentric training, and although not statistically significant, increases in PA after eccentric training.

Despite a significant increase in MVC from pre- to post-training only for EC (Fig. 4C), no significant difference in the magnitude of the change was evident between groups (Fig. 4D). Individual differences in the power output in the EC training may be a factor for the variability. The 35% increase in power output observed throughout EC training sessions was smaller than the increase in power output (3–fourfold) observed following continuous EC in a previous study (Lastayo et al 2000). It should be noted that previous studies have typically started with a low power output (~ 100 W) to avoid muscle damage and soreness (Lastayo et al. 2000; Leong et al. 2013). However, in the present study, participants were familiarized with EC before the commencement of training and thus started from a higher power output. In contrast to the previous studies reporting an increase in MVC of 13–60% after continuous EC (Lastayo et al. 1999, 2000, 2003; Lewis 2012), HIIT EC in the present study increased MVC by 12.5 ± 13.3% (− 1.4 to 34%). While these results might appear to be influenced by sample size, there were 4 participants in HIIT EC who showed more than 10% increase in MVC strength, while only 2 participants in HIIT CC showed more than 10% increase.

It was found that parameters associated with power production (PPO_10s_ and SJ) showed greater increases after EC than CC (Fig. 4). The increases in jump height confirm previous findings of 8 and 6.5% increase in CMJ height after 6 weeks of EC training of basketball players and skiers, respectively (Lindstedt et al. 2002; Gross et al. 2010). The negative trend in CMJ change in the CC group might indicate that the CMJ was not influenced by muscle function butby connective tissue function. It may be that HIIT EC improved force transfer and usage of the stretch–shortening cycle during jumps, which may have contributed to the larger increase (27%) in PPO_10s_ after EC when compared with CC (9%) (Fig. 4B). It is important to note that PPO_10s_ was measured in concentric cycling. The larger increase in PPO_10s_ suggests that specificity of the mode of muscle action is secondary for improving muscle function. It may be that cyclists can benefit from including HIIT EC training in their training routine to improve their performance. A moderate relationship was evident between the increase in the EC training intensity and CSA only (Table 1). It is interesting that greater increases in training intensity did not necessarily produce greater changes in MVC and other variables. This suggests that changes in the variables after the EC training were not necessarily determined by its training intensity, and further highlights the unique aspects of eccentric cycling training in comparison to other modes of eccentric training that utilise higher loads. It is possible that not only intensity but also the volume of eccentric contractions affects muscle adaptations observed after lower intensity eccentric exercise training.”

There were several limitations in the study. First, the number of participants in each group was not large, and only men were included in the present study. The sample size needs to be increased in the future studies and female participants, older individuals, and cyclists or other athletes should be included. The smaller sample size also means that the results should be interpreted with caution, particularly in relation to how individual differences in skill and coordination during eccentric cycling are expressed. This could be even greater at the higher intensities that were chosen for the present study. Second, as rating of perceived effort and not exertion was utilised in this study, it may make it harder to compare these results with other studies. This method was chosen to allow a more valid comparison between eccentric and concentric cycling by allowing for the utilisation of the higher power outputs one can produce during EC. As this approach was successful (Fig. 1), future studies should replicate this approach of matching exercise intensity between eccentric and concentric cycling, as well as between various EC protocols. Lastly, the participants were balanced across groups and matched via VO_2peak_ because the range of their aerobic capacity was quite large. While the aim of this study was to analyse such a general population that is active, there could be different reactions to HIIT EC within smaller participant clusters, e.g. it is possible that less fit individuals with weaker lower limbs benefit more from HIIT EC than well trained individuals. Further studies should investigate specific clusters of individuals, particularly when looking at active and trained people.

In summary, HIIT EC training performed twice a week for 8 weeks induced greater increases in quadriceps muscle CSA, PPO_10s_, and SJ when compared with HIIT CC training, and the increases in aerobic capacity were similar between the two training modalities. These results extend the previously shown benefits of HIIT EC on parameters related to muscle strength and size, as well as those related to producing high power outputs quickly (Paulsen et al. 2019; Mavropalias et al. 2022). The short time commitment required to achieve these results, especially the large (27%) improvement in peak power output, might be of particular interest for health, exercise and medical practitioners (e.g., exercise physiologists, physiotherapists and physical therapists) with recent highlights pointing out the need for training the ability to produce force quickly in our continuously aging population (Tschopp et al. 2011; Radaelli et al. 2022). Furthermore, this was the first study to highlight that increases in training intensity of EC could contribute to improvements in cardiovascular parameters, which so far was considered not possible with the generally low cardiovascular load of EC, although it would be interesting to consider this HIIT eccentric protocol in individuals with very low cardiovascular fitness. Thus, HIIT EC appears to be an effective training method for improving strength and cycling performance simultaneously. Future studies should investigate the different effects between EC and various other forms of eccentric exercise on strength and endurance parameters, compare various combinations of training parameters within and between HIIT and continuous EC, as well as consider the potential effects of several individual differences. 

## Abbreviations

CC: Concentric cycling
CMJ: Counter movement jump
CSA: Quadriceps cross-sectional area
EC: Eccentric cycling
FL: Fascicle length
HIIT: High-intensity interval training
MVC: Maximal voluntary isometric contraction
PA: Pennation angle
PPO_inc_: Incremental peak power output
PPO_10s_: Concentric sprint peak power output
SJ: Squat jump
VO_2peak_: Peak oxygen consumption
6 MW: 6-Minute walking
